# An Immune Model to Predict Prognosis of Breast Cancer Patients Receiving Neoadjuvant Chemotherapy Based on Support Vector Machine

**DOI:** 10.3389/fonc.2021.651809

**Published:** 2021-04-27

**Authors:** Mozhi Wang, Zhiyuan Pang, Yusong Wang, Mingke Cui, Litong Yao, Shuang Li, Mengshen Wang, Yanfu Zheng, Xiangyu Sun, Haoran Dong, Qiang Zhang, Yingying Xu

**Affiliations:** ^1^Department of Breast Surgery, The First Affiliated Hospital of China Medical University, Shenyang, China; ^2^Department of Breast Surgery, Cancer Hospital of China Medical University, Liaoning Cancer Hospital & Institute, Shenyang, China

**Keywords:** neoadjuvant chemotherapy, prediction, immunity, breast cancer, support vector machine

## Abstract

Tumor microenvironment has been increasingly proved to be crucial during the development of breast cancer. The theory about the conversion of cold and hot tumor attracted the attention to the influences of traditional therapeutic strategies on immune system. Various genetic models have been constructed, although the relation between immune system and local microenvironment still remains unclear. In this study, we tested and collected the immune index of 262 breast cancer patients before and after neoadjuvant chemotherapy. Five indexes were selected and analyzed to form the prediction model, including the ratio values between after and before neoadjuvant chemotherapy of CD4^+^/CD8^+^ T cell ratio; lymphosum of T, B, and natural killer (NK) cells; CD3^+^CD8^+^ cytotoxic T cell percent; CD16^+^CD56^+^ NK cell absolute value; and CD3^+^CD4^+^ helper T cell percent. Interestingly, these characters are both the ratio value of immune status after neoadjuvant chemotherapy to the baseline. Then the prediction model was constructed by support vector machine (accuracy rate = 75.71%, area under curve = 0.793). Beyond the prognostic effect and prediction significance, the study instead emphasized the importance of immune status in traditional systemic therapies. The result provided new evidence that the dynamic change of immune status during neoadjuvant chemotherapy should be paid more attention.

## Introduction

Breast cancer (BC) has been the most common cancer in women, giving rise to 30% of new cases (1). Although the overall mortality of BC is second to lung cancers, it has been the first leading cause of cancer death among females aged 20 to 59 years. With the improved treatment strategies including endocrine therapy, targeted therapy, radiotherapy, and chemotherapy, the mortality of BC declines significantly. However, the descent slowed from previous years in contrast to the accelerating decline of lung cancer and melanoma, which may be owing to a wake of immune therapy for advanced cancers. Since ipilimumab was approved by the Food and Drug Administration in 2011 (2), immune checkpoint inhibitors have become the promising therapy strategy in the past decades. As a result of low somatic mutation burden, BC showed poor response to immune therapy, traditionally regarded as an immune desert (3). Thus, the diagnosis and treatment of BC have gotten stuck in a bottleneck.

Surprisingly, immune checkpoint inhibitors were proven to function on some certain subpopulation of BC (4, 5). Atezolizumab, a programmed death 1 ligand (PD-L1) inhibitor, was suggested to prolong of overall survival (OS) in advanced triple-negative BC (TNBC) patients whose PD-L1 expressed positive. Despite the low immunogenicity, some BC patients could receive benefit from combination treatment of immune therapy and chemotherapy. Thus, the cold tumor is likely to be turned into hot tumor when treated with traditional therapeutic approaches, such as chemotherapy, radiotherapy, and targeted therapy (6). Therefore, the immune status before and after chemotherapy should be clear to unveil the key indexes that work against the malignant progression and indicate outcomes.

The increasing improvement of BC outcome is in virtue of early diagnosis, and various predictive models emerge as the times require. Clinical characteristics, including grades, TNM stages, and lymph node invasion, are essential prognostic factors besides imaging examination and have been widely used for the diagnosis and treatment (7, 8). During the past years, high-throughput sequencing made it possible to reveal the landscape of cancer transcriptome and genome. Groups of proteins, transcripts, and genes were screened to formulate new types of prediction models. The support vector machine (SVM) is a supervised learning algorithm that can achieve binary classification by linear or non-linear decision boundary. A relatively accurate maximum-margin hyperplane could be trained, even though the sample size is small. These years, many predictive models were directly constructed by SVM using high-dimensional profiles, as there are various public datasets concluding the number of presented samples, clinical information, and follow-up information (9, 10). However, genome and transcriptome data of tumor tissue samples can only reflect regional microenvironment status (11). Compared with local immune infiltration detected on sample, peripheral blood examination is much more accessible.

Hence, this study retrospectively enrolled 262 women with BC, collecting immune function indexes before and after neoadjuvant chemotherapy (NAC). And we performed univariate analysis to select independent indicators and used SVM to train a model that can predict prognosis of patients, named as NeoAdjuvant Therapy Immune Model (NATIM).

## Materials and Methods

### Patients and Preprocessing

**Figure 1:**
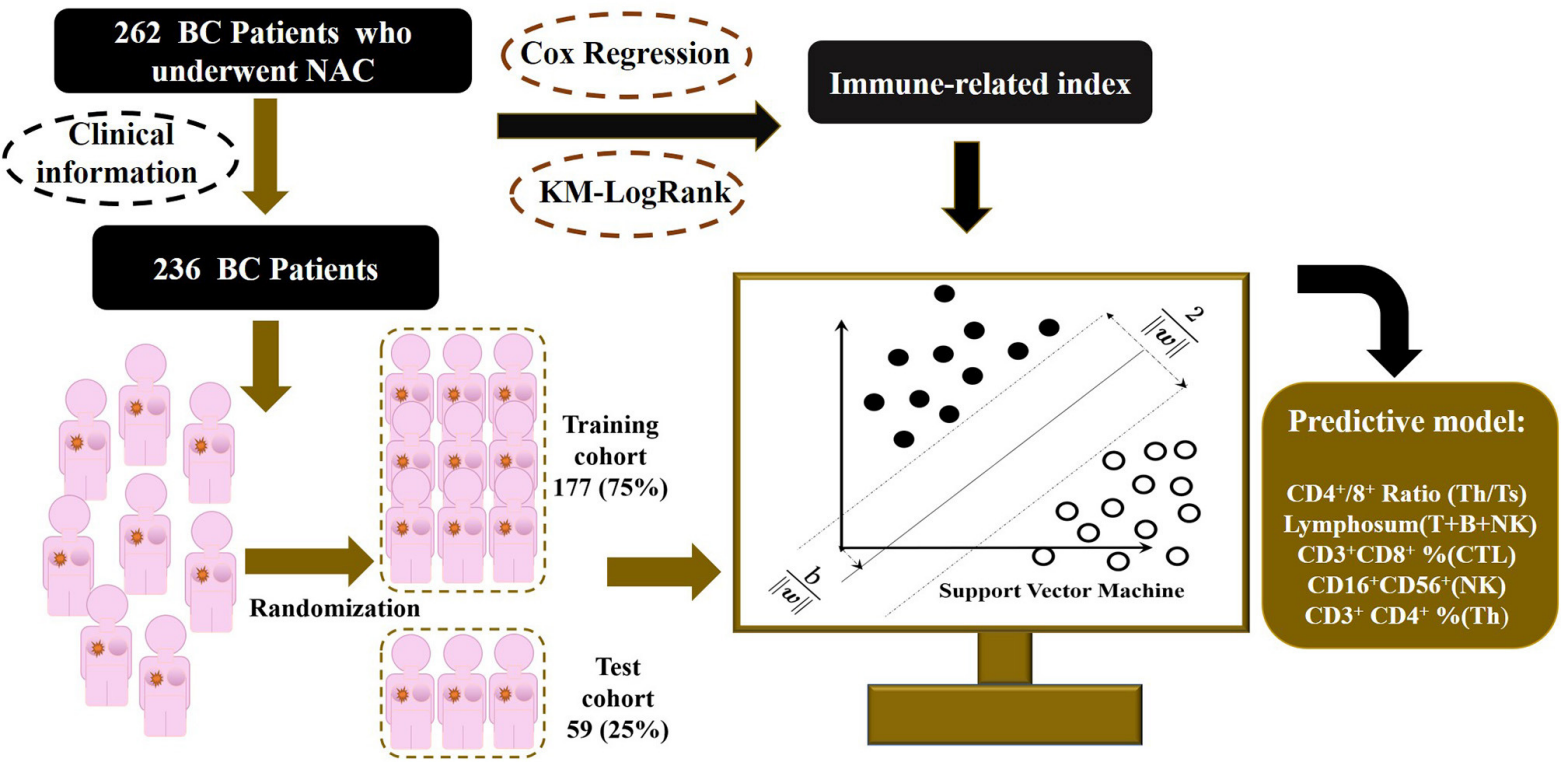
Outline of the SVM-NATIM model flow. The study enrolled 262 women with breast cancer, collecting immune function indexes before and after neoadjuvant chemotherapy. After data processing, 236 patients were put into modeling procedure. Univariate analysis and supporting vector machine were performed to select independent indicators and train a predictive model, named as NeoAdjuvant Therapy Immune Model (NATIM).

The flowchart of the study is shown in Figure 1. The study has been approved by the Ethics Committee of the Cancer Hospital of China Medical University. As shown in Figure 1, the total cohort included 262 patients from the Breast Surgery Department of Cancer Hospital of China Medical University who received NAC during the period of 2014 and 2018. The clinical and pathological features were collected as follows: age; gender; grade; clinical primary tumor (T) and regional nodes (N) stage at diagnosed; grade; pathological T and N stage at surgery; estrogen receptor (ER), progesterone receptor (PR), human epidermal growth factor receptor (HER2), and Ki67 percentage before and after NAC; Miller–Payne (MP) grade; and therapeutic plans of NAC. The histopathological diagnosis and histochemical examination were performed on tumor biopsy before NAC and tumor specimens at surgery after NAC, and TNM stage followed the eighth edition of AJCC TNM staging. The follow-up data including death and date were collected every 6 months by telephone and OS was calculated from the date of surgery to the date of death or the latest follow-up.

Patients whose information was missed had been excluded, resulting in a total of 236 patients enrolled finally. To minimize the bias, outliers were assessed and winsorized. Characteristics that were unknown for more than 10% of overall patients had been deleted, whereas missed items that remained were assigned as average values.

### Immune Status of Patients

All immune-related indexes in peripheral blood that reflected lymphocytic immune function were examined by clinical laboratory of Cancer Hospital of China Medical University before and after NAC. Immune-related indexes include CD4^+^/CD8^+^T cell ratio, CD16^+^CD56^+^ natural killer (NK) cell percent, CD16^+^CD56^+^ NK cell absolute value, CD19^+^ B cell percent, CD19^+^B cell absolute value, CD3^+^ T cell percent, CD3^+^ T cell absolute value, CD3^+^CD4^+^ helper T cell percent, CD3^+^CD4^+^ helper T cell absolute value, CD3^+^CD8^+^ cytotoxic T cell percent, CD3^+^CD8^+^ cytotoxic T cell absolute value, CD45^+^ T cell absolute value, and lymphosum of T, B, and NK cells.

### Statistical Programs and Software

Statistical analyses were performed with R version 3.5.3 and SPSS version 19. The SVM algorithm was built using the LIBSVM program 27 based on MATLAB 2017a (MathWorks), and the source code was uploaded to Github (https://github.com/zjslp218/NATIM-SVM-model).

## Results

### Change of Characteristics of Peripherally Immune Status Before and After NAC

We collected the information of 262 BC patients who received NAC before surgery (Table 1) and sorted out 236 patients whose clinical characteristics and immune function examination results before and after NAC were both accessible. As shown in Tables 2, 3, after NAC, CD4^+^/CD8^+^ T cell ratio elevated to 7.01 ± 72.19 from 1.95 ± 0.85 and lymphosum of T, B, and NK cell reached to 216.28 ± 750.71 from 128.7 ± 326.24. On the contrary, CD16^+^CD56^+^ NK cell absolute value, CD19^+^ B cells, and CD45^+^ T cells were decreased, among which CD19^+^ B cell absolute value and percent decreased most.

**Table 1:** Clinicopathological characteristics of patients when diagnosed.

**Characteristics**	**Number**	**Percent (%)**
All patients	262	100
**Age, years**		
≤50	104	39.7
>50	158	60.3
**Grade**		
1	3	1.1
2	118	45.0
3	14	5.3
Unknown	127	48.5
**cT**		
1	3	1.1
2	220	84.0
3	21	8.0
Unknown	18	6.9
**N**		
–	159	60.7
+	14	5.3
Unknown	89	34.0
**ER**		
–	104	39.7
+	145	55.3
Unknown	13	5.0
**PR**		
–	150	57.2
+	99	37.8
Unknown	13	5.0
**HER2**		
–	147	56.1
+	63	24.0
Unknown	52	19.9
**Ki67, %**		
≤20	57	21.7
>20	192	73.3
Unknown	13	5.0
**Subtype**		
Luminal A	35	13.4
Luminal B	81	30.9
Her2 positive	45	17.2
TNBC	49	18.7
Unknown	52	19.8
**NAC**		
Anthracycline- and taxane-based	238	90.8
Taxane-based only	22	8.4
Unknown	2	0.8
**MP grade**		
1	11	4.2
2	87	33.2
3	103	39.3
4	39	14.9
5	12	4.6
Unknown	10	3.8

*ER, estrogen receptor; PR, progesterone receptor; HER2, human epidermal growth factor receptor; TNBC, triple-negative breast cancer; NAC, neoadjuvant chemotherapy; MP, Miller–Payne*.

**Table 2:** Statistical distribution of immune function indexes before NAC.

	**Average ± SD**	**Range**	**Percentage**		
			**25%**	**Median**	**75%**
CD4^+^/CD8^+^ T cell ratio	1.95 ± 0.85	0.6–4.99	1.27	1.82	2.37
CD16^+^CD56^+^ NK cell percent	20.68 ± 8.1	4.03–48.03	15.15	19.36	25.46
CD16^+^CD56^+^ NK cell absolute value	465.25 ± 290.26	24.09–1,655.28	287.08	383.74	603.43
CD19^+^ B cell percent	10.92 ± 10.32	2.17–157.59	7.58	9.63	12.77
CD19^+^B cell absolute value	223.66 ± 128.89	14.22–975.57	134.52	197.00	278.00
CD3^+^ T cell percent	71.28 ± 48.98	6.44–802.65	63.65	69.62	74.50
CD3^+^ T cell absolute value	1,532.19 ± 938.8	43.62–9,479.66	1, 055.26	1, 314.25	1, 752.82
CD3^+^CD4^+^ helper T cell percent	55.65 ± 144.9	19.54–1,750.31	34.67	40.95	45.60
CD3^+^CD4^+^ helper T cell absolute value	900.42 ± 636.94	23.15–6,294.57	584.80	761.20	1, 033.00
CD3^+^CD8^+^ cytotoxic T cell percent	33.42 ± 87.46	7.51–1,076.42	18.56	23.52	28.71
CD3^+^CD8^+^ cytotoxic T cell absolute value	539.65 ± 412.78	7.77–4,121.23	328.50	471.28	613.45
CD45^+^ T cell absolute value	2,243.73 ± 1,235.11	478.1–11,715.49	1, 587.53	1, 984.50	2, 519.49
Lymphosum of T, B, and NK cells	128.7 ± 326.24	30.24–4,321.96	99.53	99.77	99.86

*NK cell, natural killer cell*.

**Table 3:** Statistical distribution of immune function indexes after NAC.

	**Average ± SD**	**Range**	**Percentage**		
			**25%**	**Median**	**75%**
CD4^+^/CD8^+^ T cell ratio	7.01 ± 72.19	7.01–72.19	7.01	72.19	7.01
CD16^+^CD56^+^ NK cell percent	364.14 ± 5, 289.26	364.14–5,289.26	364.14	5, 289.26	364.14
CD16^+^CD56^+^ NK cell absolute value	346.03 ± 279.22	346.03–279.22	346.03	279.22	346.03
CD19^+^ B cell percent	5.13 ± 18.66	5.13–18.66	5.13	18.66	5.13
CD19^+^B cell absolute value	69.78 ± 74.85	69.78–74.85	69.78	74.85	69.78
CD3^+^ T cell percent	78 ± 28.68	78–28.68	78.00	28.68	78.00
CD3^+^ T cell absolute value	1,330.41 ± 952.48	1,330.41–952.48	1, 330.41	952.48	1, 330.41
CD3^+^CD4^+^ helper T cell percent	50.61 ± 113.48	50.61–113.48	50.61	113.48	50.61
CD3^+^CD4^+^ helper T cell absolute value	2,322.06 ± 23,648.54	2,322.06–23,648.54	2, 322.06	23, 648.54	2, 322.06
CD3^+^CD8^+^ cytotoxic T cell percent	27.45 ± 8.67	27.45–8.67	27.45	8.67	27.45
CD3^+^CD8^+^ cytotoxic T cell absolute value	498.25 ± 406.11	498.25–406.11	498.25	406.11	498.25
CD45^+^ T cell absolute value	1,748 ± 1,187.9	1,748–1,187.9	1, 748.00	1, 187.90	1, 748.00
lymphosum of T, B, and NK cells	216.28 ± 750.71	216.28–750.71	216.28	750.71	216.28

*NK cell, natural killer cell*.

The relationship of peripherally immune status before and after NAC and pathological indexes when first diagnosed were additionally assessed (Supplementary Tables 1–3). The change of CD3^+^ T cell percent (after NAC vs. baseline) was significantly increased in ER-positive subgroup when compared to ER-negative subgroup (1.26 ± 1.05 vs. 1.18 ± 0.93, *P* = 0.031). In addition to CD3^+^ T cell percent (1.33 ± 1.28 vs. 1.16 ± 0.78, *P* = 0.023), the change of CD16^+^CD56^+^ NK cell percent (after NAC vs. baseline) circulating in periphery blood was much in the PR-positive subgroup than in the PR-negative subgroup (52.67 ± 485.31 vs. 1.02 ± 0.3, *P* = 0.048). Dissimilarly, the ratio of CD3^+^CD4^+^ helper T cell percent and CD19^+^ B cell percent after NAC to that of baseline was less in HER2-positive subpopulation rather than HER2-negative subpopulation (CD3^+^CD4^+^ helper T cell percent, 1.01 ± 0.26 vs. 1.34 ± 3.14, *P* = 0.019; CD19^+^ B cell percent, 0.38 ± 0.33 vs. 0.61 ± 2.68, *P* = 0.046). Above distribution and change intimated the different immune cell populations evoked by NAC in peripheral blood.

Then, we analyzed correlation between the peripheral immune indexes before and after NAC (Supplementary Figures 1, 2). Spearman correlation was performed, and it suggested that CD45^+^ T cell absolute value, CD3^+^ T cell absolute value, and CD3^+^CD4^+^ helper T cell absolute value were strongly related to each other positively. And CD16^+^CD56^+^ NK cell absolute value was negatively related to CD3^+^ T cell percent.

### Selection of Related Immune Index

**Figure 2:**
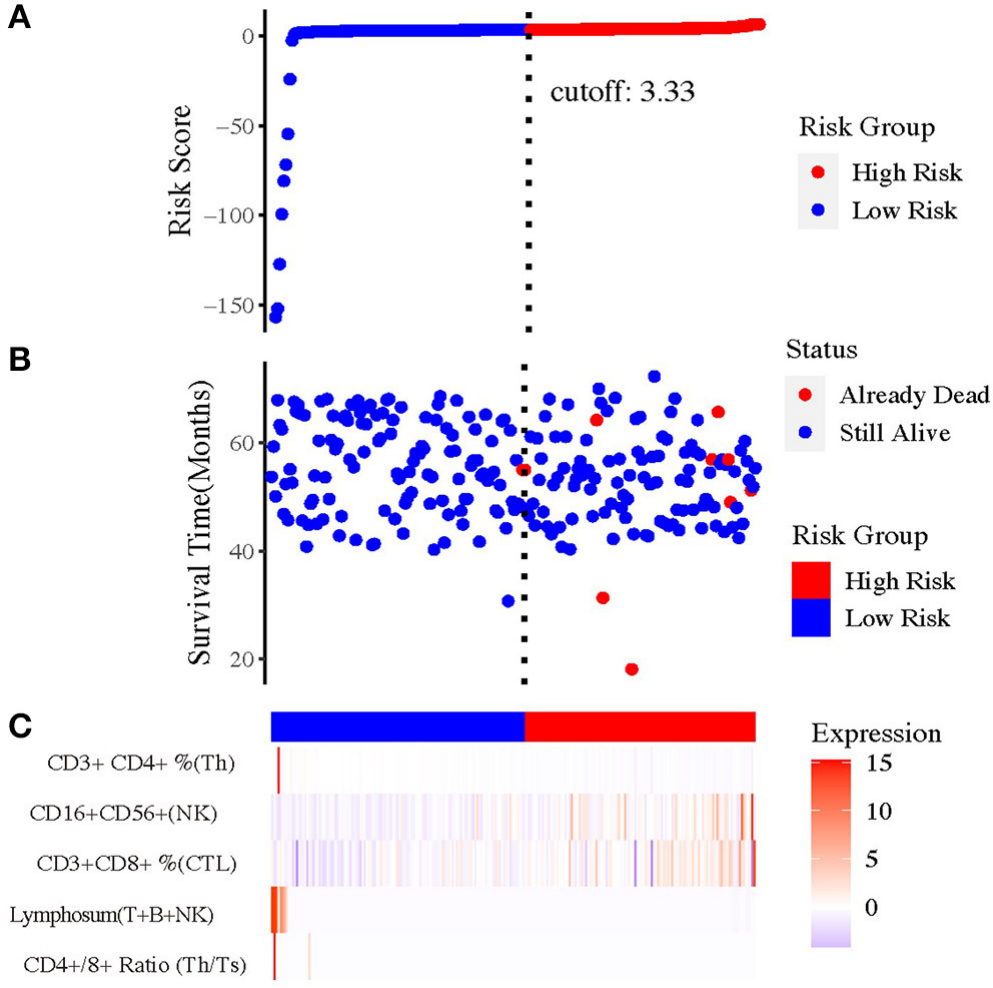
The prognostic value of risk model of the five immune-related indexes. **(A)** The risk curve based on the model with the largest area under curve (AUC). **(B)** The scatterplot based on the survival status of each sample. The blue and red plots represent low risk and high risk, respectively. **(C)** The heatmap showed the enrichment level of immune-related indexes in peripheral blood in high- and low-risk subgroups.

To select the most appropriate immune function indexes, we calculated the ratio of each immune function index after NAC to the baseline and put them as independent indexes, besides the direct value before and after NAC. To distinguish the three values of each index, the values before and after NAC were named as Index(b) and Index(a), whereas the ratio values were Index(a/b) below. Subsequently, we performed Cox regression and Kaplan–Meier (KM) analysis on all lymphocytic immune function indexes in peripheral blood for univariate analysis (Supplementary Figures 3B–F). And forest plot was drawn and inferred that the indexes (a/b) showed overall better interaction with prognosis (Supplementary Figure 4). Under help of SVM, the most optimal combination that consisted of five indexes were sorted out (Figure 2), including CD4^+^/CD8^+^ T cell ratio (a/b); lymphosum of T, B, and NK cells (a/b); CD3^+^CD8^+^ cytotoxic T cell percent (a/b); CD16^+^CD56^+^ NK cell absolute value (a/b); and CD3^+^CD4^+^ helper T cell percent (a/b).

### Construction and Evaluation of the NATIM

**Figure 3:**
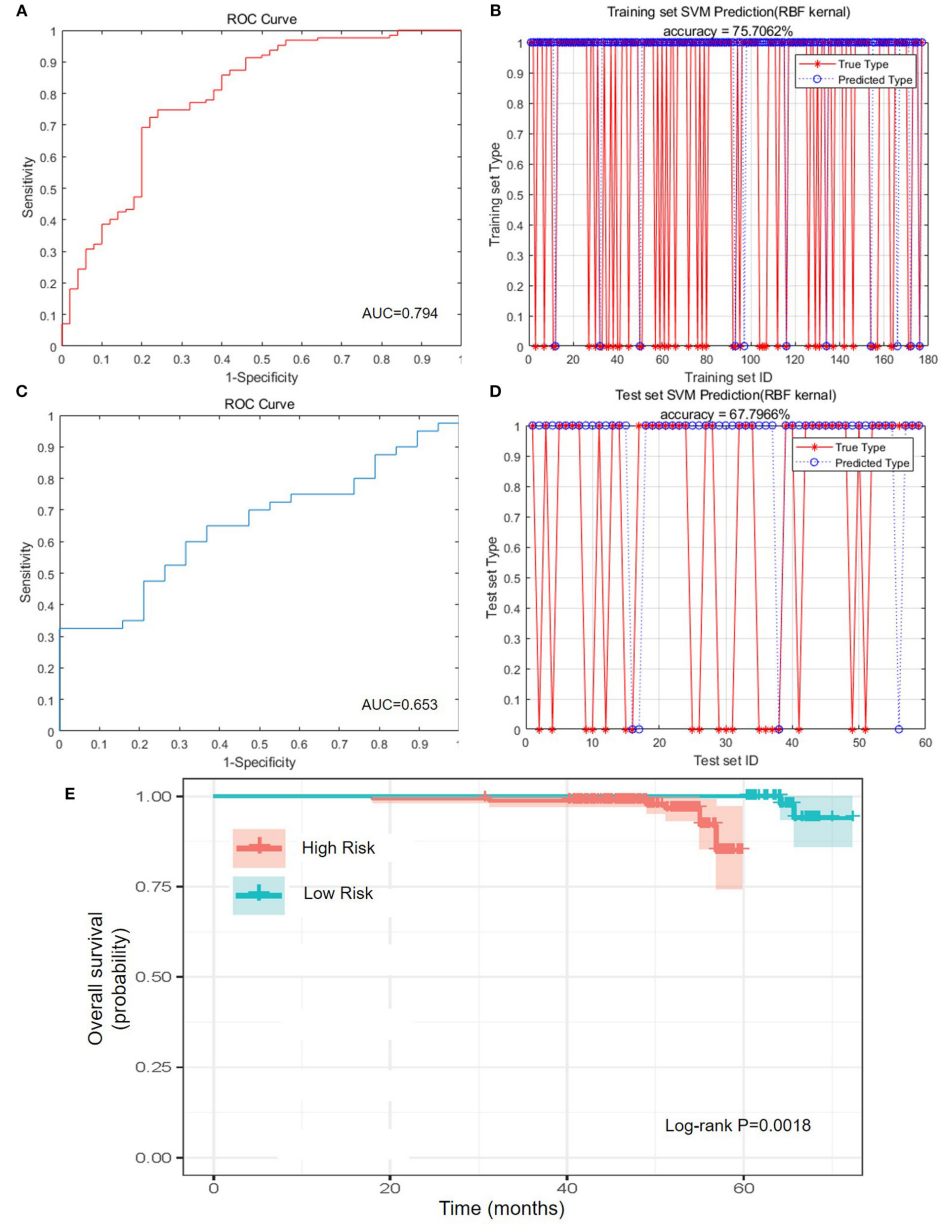
Predictive efficacy of NATIM. **(A)** Receiver operating characteristic (ROC) curve and area under curve (AUC) of NeoAdjuvant Therapy Immune Model (NATIM) in training cohort. **(B)** Prediction accuracy of NATIM in training cohort. **(C)** ROC and AUC of NATIM in test cohort. **(D)** Prediction accuracy of NATIM in test cohort. **(E)** Kaplan–Meier plot of NATIM between high- and low-risk population.

To better classify the patients with different prognosis, the population was divided into two subgroups by the comprehensive assessment of live status and OS. Those who lived for more than 5 years were assigned as low-risk population, whereas those who were dead within 5 years or lived for <5 years were assigned as high-risk population. Because of the lack of external validation cohort, training cohort and test cohort were randomly selected and formed by division of original cohort. After training of training set and adjustment of parameters, SVM was applied to construct the best-performing model with Gaussian kernel. The accuracy reached 75.71% (134/177) in the training set, and the area under curve (AUC) reached 0.794, highlighting the well-prognostic effectiveness of NATIM (Figures 3A,B). Then we used randomized testing cohort to test the efficacy and obtained an accuracy of 67.80% (40/59) and AUC of 0.653 in the testing cohort (Figures 3C,D). Furthermore, the KM plot was shown to validate the effective of NATIM to classify the high- and low-risk subpopulation (*P* = 0.0018) (Figure 3E).

Therefore, we drew the receiver operating characteristic curve and calculated the AUC of each single immune index (Supplementary Figure 3A). All the AUCs of single immune indexes were lower than that of NATIM (*P*-value of CD4^+^/CD8^+^ T cell ratio; lymphosum of T, B, and NK cell; and CD3^+^CD8^+^ cytotoxic T cell percent were both <0.05). Accordingly, the above results claimed that NATIM can provide an independent approach to predict the prognosis, more effective than any single immune cell model.

## Discussion

In recent decades, immune therapy has become the most promising strategy. Since reaching several peaks that contributed by clinical and preclinical breakthroughs, progresses against BC slow down. Distinct from other metastatic cancers including non-small cell lung cancer, melanoma, and gastric cancer, BCs react inertly to systemic and local immune mobilization. In TONIC trial (NCT02499367), 67 patients who were diagnosed as having advanced TNBC randomly received a 2-week inducible therapy and sequenced by three cycles of nivolumab, a programmed death 1 (PD-1) inhibitor (12). Surprisingly, doxorubicin and cisplatin were found to induce T cell infiltration and subsequently acquire the highest clinical response rate. Afterward, researches about the effect of traditional treatment on microenvironment came out one after the other. Chemotherapy was proved to impact individual resistance to different types of drugs by activating, recruiting, and polarizing tumor-related immune cells in addition to immunogenic cell death (13). Chemotherapy could directly kill immunosuppressive cells and effective cells, increasing infiltration of tumor-related macrophages and then induced drug resistance (14, 15). The dual effect of chemotherapy on immunity leaves the mechanism complex and potential to be targeted as diagnostic and therapeutic markers. Our results showed that CD4^+^/CD8^+^ T cell ratio increased from immune suppressive status to an active status, indicating an elevated neoantigen-recognitive and killing capacity of regional immune cells.

Outcome prediction and treatment benefit models relied on clinical features as mainly elements were variously developed and validated around the 20th century (16). With the rapid development of next-generation sequencing and single cell sequencing, genome and transcriptome of cancer patients have been profiled accurately. Diverse models and biomarkers have been built up to describe and predict immune status, drug response, and prognosis (17–19). Shao et al. analyzed transcriptional expression atlas of TNBC, selected eight mRNAs and two lncRNAs, and constructed a predictive model that can forecast chemotherapy response and outcome of TNBC patients based on the above 10 transcripts (20). A 13-epigenetic characteristics were also formed as a model to distinct low- and high-risk BC population, along with the transcription (21). Moreover, distant metastatic sites of TNBC could be well-predicted by eight signatures in paraffin-embedded tissues likewise (22). However, immunity includes not only microenvironment surrounding the tumor cells, but also the peripherally immune cells that reflect the systemic immunity. Supervision of immune components of peripheral blood is unneglectable. Axelrod et al. performed single cell sequencing on PD-1–high CD8^+^ T cells in peripheral blood along with the exploration on tumor immune microenvironment of tumor tissues from advanced BC patients who ever received NAC (23). The result at the genetic level suggested the opposite status of peripheral blood and local immune microenvironment.

We collected 262 patients and finally enrolled 236 BC patients who underwent immune function examination in peripheral blood before and after NAC. KM log rank and Cox regression were adopted for the univariate analysis. Three dynamic indexes that reflect changes caused by NAC, CD4^+^/CD8^+^ T cell ratio (a/b), CD3^+^CD8^+^ cytotoxic T cell percent (a/b), and lymphosum of T, B, and NK cells (a/b) were proven to be an effective predictive factor. Then, we randomly divided the cohort into training cohort and validation cohort and used SVM to train the best model, which arrives at an accuracy of 0.75. SVM is an important kind of machine learning algorithm regarded as the best classifier suitable for training sets whose sample size is too small. SVM is a generalized linear regression model for linear subscenarios. And for non-linear subscenarios, the samples of low-dimensional feature space could be mapped to high-dimensional space by nuclear technique to achieve linear analysis of non-linear samples. The theoretical basis of the SVM method is non-linear mapping by using kernel functions instead of non-linear mapping to high-dimensional space. In addition, the optimization goal of SVM is to minimize the structured risk instead of the empirical risk, avoiding the problem of overfit. Then it got the structured description of the data distribution through the margin concept, reducing the requirements of data size and data distribution, leading excellent generalization ability. Consequently, SVM can get more accurate results on small sample training sets than other algorithms.

Neoadjuvant therapy is an appropriate period to evaluate the change of immune status caused by chemotherapy, avoiding the traumatic immune response caused by any other treatment including operation. Furthermore, patients who undergo neoadjuvant therapy are at earlier stages with an improved immunity rather than those who are at advanced stages. Additionally, peripheral blood examination is much easier and cheaper to perform for both doctors and patients, which is an important element for a well-used predictive model. It is worth noting that the indexes sorted by regression with best distinction for prognosis are both the ratio value of immune status after NAC to the baseline. The used studies always studied instantaneous status of immune function of cancer patients, but our results first proved that the dynamic change of immune function may demonstrate much more clues.

This study still has some limitations. First, the immune function assessment of peripheral blood was just carried out in the last few years. Clinical cohort with entire immune examination before and after NAC is so rare that external validation is lacking. Hence, more prospective researches or large-scale studies are urgently required to affirm this result. Second, owing to the specificity of data, the overall patients who enrolled are still not adequate enough to be divided for subgroup analysis. Most immune-related clinical experiments focus attention on TNBC or advanced patients in consideration of ethics. However, the systemic and local immune status of each subtype of BCs ought to be distinct to each other and should be profiled accurately. Finally, the present study just states the peripheral other than local microenvironment immune status. Therefore, it would be better to compare the immune status both from peripheral blood and microenvironment correspondingly and describe the systemic and local immune characteristics exactly before and after chemotherapy. We will enlarge the data and update the model in the future.

In conclusion, we constructed a new immune index model of BC by integrating immune cell absolute value and percentage of dissimilar immune cell population. Our peripheral immune index model is practical for predicting the prognosis of BC patients who received NAC. Further studies are warranted to validate these results.

## Data Availability Statement

The raw data supporting the conclusions of this article will be made available by the authors, without undue reservation.

## Author Contributions

MoW wrote the manuscript. YW and MeW studied R-related data and process. LY and HD performed SPSS-related process. XS revised the language. ZP and SL was in charge of the data collecting, while MC and YZ enrolled the patients. YX and QZ designed the study. All authors contributed to the article and approved the submitted version.

## Conflict of Interest

The authors declare that the research was conducted in the absence of any commercial or financial relationships that could be construed as a potential conflict of interest.

## Supplementary Material

Supplementary Figure 1 Spearman correlation between the peripheral immune indexes before neoadjuvant chemotherapy.

Supplementary Figure 2 Spearman correlation between the peripheral immune indexes after neoadjuvant chemotherapy.

Supplementary Figure 3 Evaluation of single immune index. **(A)** ROC and AUC of single immune index; **(B)** KM plot of CD16+CD56+ NK cell absolute value (a/b); **(C)** KM plot of lymphosum of T cell, B cell and NK cell (a/b); **(D)** KM plot of CD3+ CD8+ cytotoxic T cell percent (a/b); **(E)** KM plot of CD4+/CD8+ T cell ratio (a/b); **(F)** KM plot of CD3+ CD4+ helper T cell percent (a/b).

Supplementary Figure 4 Forest plot of index selected by Cox regression.

Supplementary Table 1 Relationship of peripherally immune status change before and after NAC and ER status at diagnosis.

Supplementary Table 2 Relationship of peripherally immune status change before and after NAC and PR status at diagnosis.

Supplementary Table 3 Relationship of peripherally immune status change before and after NAC and HER2 status at diagnosis.
