# Supplementary figures and images for: An Immune Model to Predict Prognosis of Breast Cancer Patients Receiving Neoadjuvant Chemotherapy Based on Support Vector Machine
Source: Front Oncol. 2021 Apr 27;11:651809. doi: 10.3389/fonc.2021.651809 (PMC8111218; doi:10.3389/fonc.2021.651809)

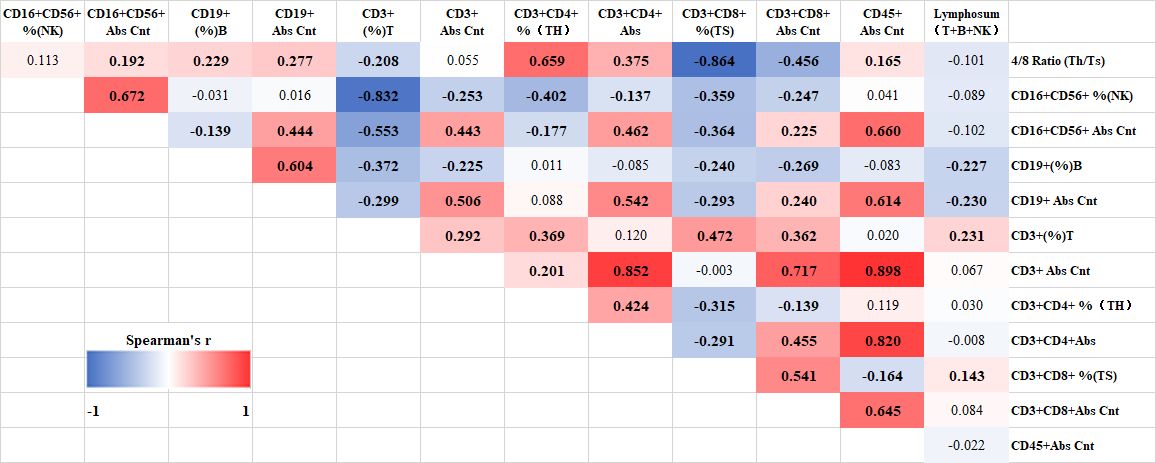

Supplement: Supplementary Figure 1 — Spearman correlation between the peripheral immune indexes before neoadjuvant chemotherapy. [file Image_1.JPEG]

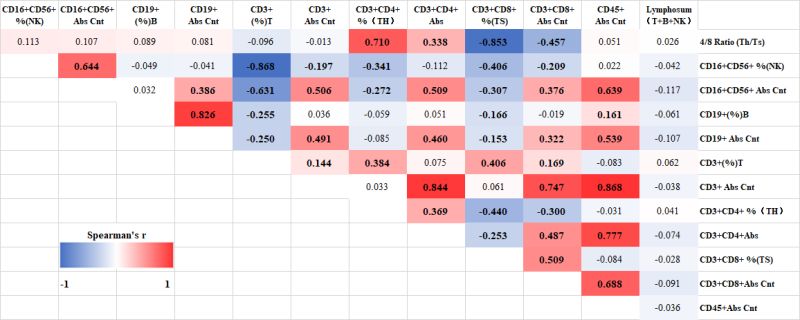

Supplement: Supplementary Figure 2 — Spearman correlation between the peripheral immune indexes after neoadjuvant chemotherapy. [file Image_2.JPEG]

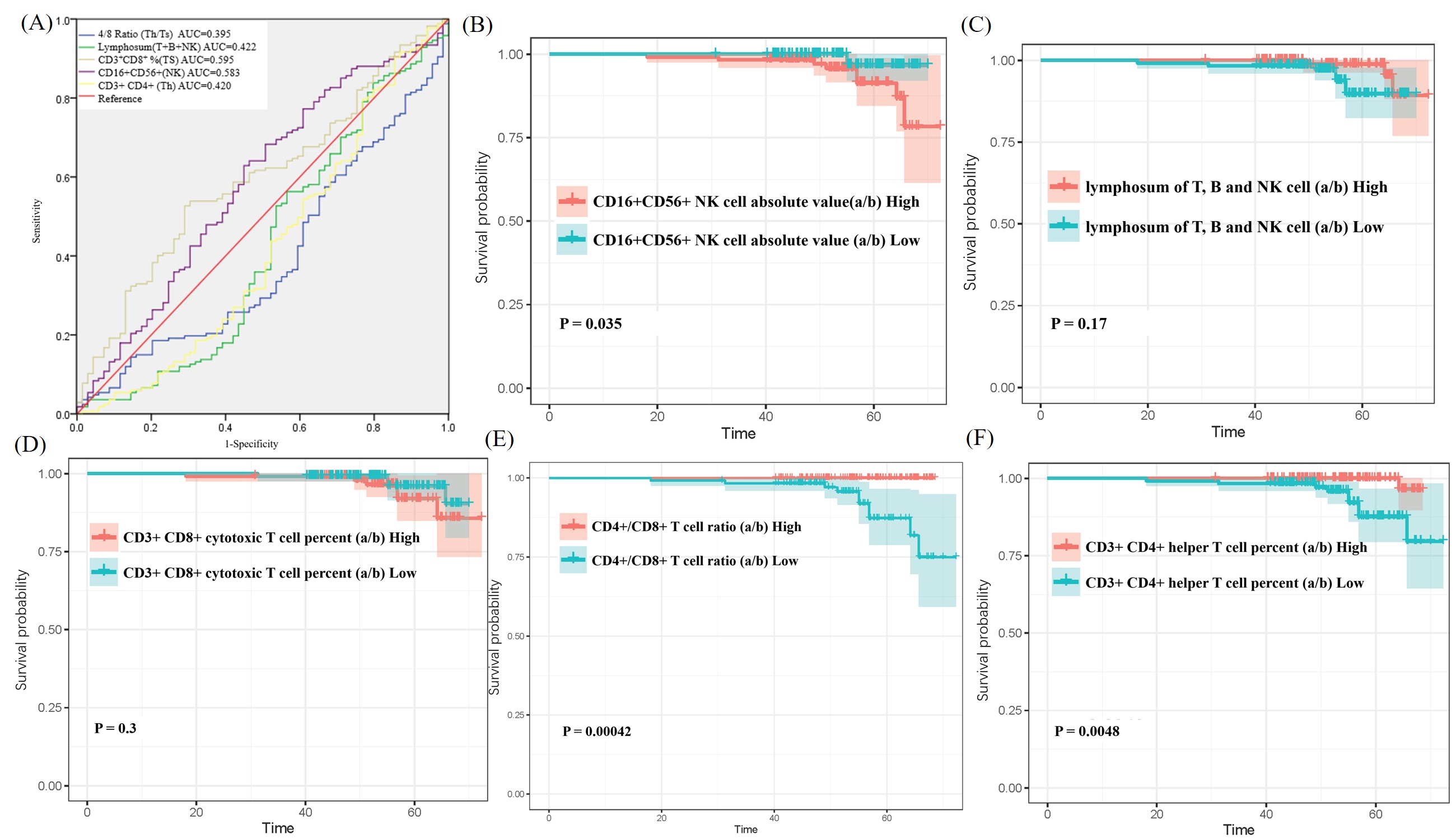

Supplement: Supplementary Figure 3 — Evaluation of single immune index. (A) ROC and AUC of single immune index; (B) KM plot of CD16+CD56+ NK cell absolute value (a/b); (C) KM plot of lymphosum of T cell, B cell and NK cell (a/b); (D) KM plot of CD3+ CD8+ cytotoxic T cell percent (a/b); (E) KM plot of CD4+/CD8+ T cell ratio (a/b); (F) KM plot of CD3+ CD4+ helper T cell percent (a/b). [file Image_3.JPEG]

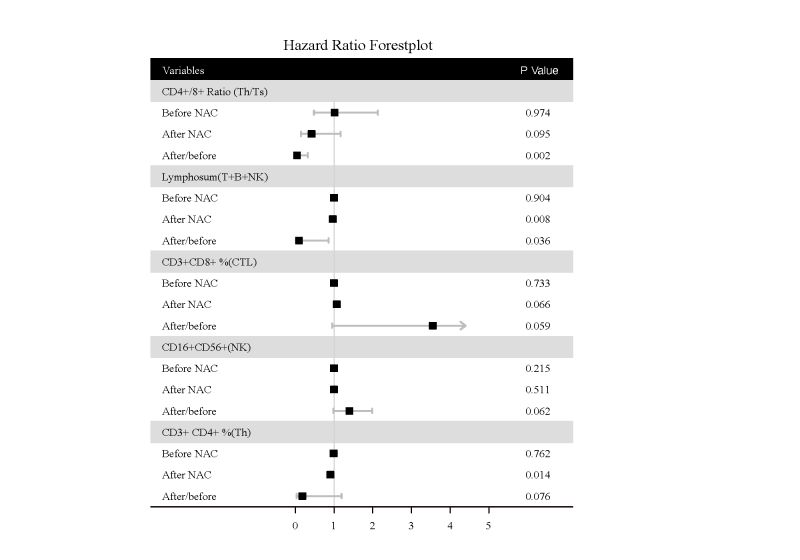

Supplement: Supplementary Figure 4 — Forest plot of index selected by Cox regression. [file Image_4.JPEG]
