# Supplementary material for: An Immune Model to Predict Prognosis of Breast Cancer Patients Receiving Neoadjuvant Chemotherapy Based on Support Vector Machine
Source: Front Oncol. 2021 Apr 27;11:651809. doi: 10.3389/fonc.2021.651809 (PMC8111218; doi:10.3389/fonc.2021.651809)
Supplement: Supplementary Table 1 — Relationship of peripherally immune status change before and after NAC and ER status at diagnosis. [file Table_1.DOCX]

Supplementary Material

# Supplementary Tables

**Supplement Table 1.** **Relationship of peripherally immune status change before and after NAC and ER status at diagnosis.**

| Characteristics change of adjuvant chemotherapy | ER | | P value |
| --- | --- | --- | --- |
|  | negative(n=104) | positive(n=132) |  |
| CD4+/CD8+T cell ratio | 10.73±99.94 | 1.72±9.03 | 0.231 |
| CD16+CD56+ NK cell percent | 1.03±0.31 | 35.45±396.25 | 0.090 |
| CD16+CD56+ NK cell absolute value | 0.92±1.02 | 0.87±0.69 | 0.920 |
| CD19+ B cell percent | 0.38±0.3 | 0.66±2.99 | 0.153 |
| CD19+B cell absolute value | 0.38±0.64 | 0.35±0.3 | 0.216 |
| CD3+ T cell percent | 1.18±0.93 | 1.26±1.05 | **0.031** |
| CD3+ T cell absolute value | 1.35±2.7 | 1.02±0.81 | 0.580 |
| CD3+ CD4+ helper T cell percent | 1.04±0.25 | 1.4±3.51 | 0.068 |
| CD3+ CD4+ helper T cell absolute value | 6.87±53.98 | 1.04±0.95 | 0.989 |
| CD3+ CD8+ cytotoxic T cell percent | 1.19±0.35 | 1.17±0.21 | 0.225 |
| CD3+ CD8+ cytotoxic T cell absolute value | 1.26±2.17 | 1.48±3.9 | 0.814 |
| CD45+ T cell absolute value | 0.83±0.47 | 0.93±0.72 | 0.348 |
| Lymphosum of T cell, B cell and NK cell | 2.74±9.41 | 1.72±5.61 | 0.494 |

Note: P value was assessed by Kruskal-Wallis tests. The values in the table were calculated as the ratio value of immune status after neoadjuvant chemotherapy to the baseline.

* Abbreviation: NK cell, natural killer cell;
