# Supplementary material for: An Immune Model to Predict Prognosis of Breast Cancer Patients Receiving Neoadjuvant Chemotherapy Based on Support Vector Machine
Source: Front Oncol. 2021 Apr 27;11:651809. doi: 10.3389/fonc.2021.651809 (PMC8111218; doi:10.3389/fonc.2021.651809)
Supplement: Supplementary Table 2 — Relationship of peripherally immune status change before and after NAC and PR status at diagnosis. [file Table_2.DOCX]

Supplementary Material

# Supplementary Tables

**Supplement Table 2.** **Relationship of peripherally immune status change before and after NAC and PR status at diagnosis.**

| Characteristics change of adjuvant chemotherapy | PR | | P value |
| --- | --- | --- | --- |
|  | negative(n=148) | positive(n=88) |  |
| CD4+/CD8+T cell ratio | 7.82±83.78 | 2.1±11.06 | 0.404 |
| CD16+CD56+ NK cell percent | 1.02±0.3 | 52.67±485.31 | **0.048** |
| CD16+CD56+ NK cell absolute value | 0.9±0.88 | 0.88±0.8 | 0.338 |
| CD19+ B cell percent | 0.38±0.29 | 0.81±3.66 | 0.107 |
| CD19+B cell absolute value | 0.37±0.56 | 0.35±0.3 | 0.283 |
| CD3+ T cell percent | 1.16±0.78 | 1.33±1.28 | **0.023** |
| CD3+ T cell absolute value | 1.24±2.29 | 1.04±0.92 | 0.987 |
| CD3+ CD4+ helper T cell percent | 1.05±0.23 | 1.56±4.29 | 0.076 |
| CD3+ CD4+ helper T cell absolute value | 5.11±45.27 | 1.08±1.1 | 0.851 |
| CD3+ CD8+ cytotoxic T cell percent | 1.18±0.32 | 1.18±0.2 | 0.521 |
| CD3+ CD8+ cytotoxic T cell absolute value | 1.24±1.9 | 1.63±4.73 | 0.809 |
| CD45+ T cell absolute value | 0.84±0.46 | 0.97±0.83 | 0.726 |
| Lymphosum of T cell, B cell and NK cell | 2.44±8.38 | 1.71±5.81 | 0.406 |

Note: P value was assessed by Kruskal-Wallis tests. The values in the table were calculated as the ratio value of immune status after neoadjuvant chemotherapy to the baseline.

* Abbreviation: NK cell, natural killer cell;
