# Supplementary material for: An Immune Model to Predict Prognosis of Breast Cancer Patients Receiving Neoadjuvant Chemotherapy Based on Support Vector Machine
Source: Front Oncol. 2021 Apr 27;11:651809. doi: 10.3389/fonc.2021.651809 (PMC8111218; doi:10.3389/fonc.2021.651809)
Supplement: Supplementary Table 3 — Relationship of peripherally immune status change before and after NAC and HER2 status at diagnosis. [file Table_3.DOCX]

Supplementary Material

# Supplementary Tables

**Supplement Table 3.** **Relationship of peripherally immune status change before and after NAC and HER2 status at diagnosis.**

| Characteristics change of adjuvant chemotherapy | HER2 | | P value |
| --- | --- | --- | --- |
|  | negative(n=165) | positive(n=71) |  |
| CD4+/CD8+T cell ratio | 7.74±79.71 | 0.93±0.31 | 0.193 |
| CD16+CD56+ NK cell percent | 28.57±354.42 | 1.01±0.3 | 0.335 |
| CD16+CD56+ NK cell absolute value | 0.86±0.66 | 0.98±1.18 | 0.630 |
| CD19+ B cell percent | 0.61±2.68 | 0.38±0.33 | **0.046** |
| CD19+B cell absolute value | 0.38±0.54 | 0.31±0.3 | 0.112 |
| CD3+ T cell percent | 1.28±1.19 | 1.09±0.16 | 0.061 |
| CD3+ T cell absolute value | 1±0.77 | 1.57±3.23 | 0.735 |
| CD3+ CD4+ helper T cell percent | 1.34±3.14 | 1.01±0.26 | **0.019** |
| CD3+ CD4+ helper T cell absolute value | 4.41±42.83 | 1.75±3.85 | 0.777 |
| CD3+ CD8+ cytotoxic T cell percent | 1.18±0.2 | 1.19±0.41 | 0.684 |
| CD3+ CD8+ cytotoxic T cell absolute value | 1.36±3.49 | 1.44±2.66 | 0.999 |
| CD45+ T cell absolute value | 0.89±0.66 | 0.88±0.53 | 0.845 |
| Lymphosum of T cell, B cell and NK cell | 2.69±8.95 | 0.97±0.16 | 0.418 |

Note: P value was assessed by Kruskal-Wallis tests. The values in the table were calculated as the ratio value of immune status after neoadjuvant chemotherapy to the baseline.

* Abbreviation: NK cell, natural killer cell;
